# Identification of key biomarkers for STAD using filter feature selection approaches

**DOI:** 10.1038/s41598-022-21760-w

**Published:** 2022-11-18

**Authors:** Yangyang Wang, Jihan Wang, Ya Hu, Jingbo Shangguan, Qiying Song, Jing Xu, Hanping Wang, Mengju Xue, Liping Wang, Yuanyuan Zhang

**Affiliations:** 1 School of Electronics and Information, Northwestern Polytechnical University, Xi’an, Shaanxi China; 2 Institute of Medical Research, Northwestern Polytechnical University, Xi’an, Shaanxi China; 3 Department of Medical College, Hunan Polytechnic of Environment and Biology, Hengyang, Hunan China; 4 Department of Basic Medicine, School of Medicine, Xi’an International University, Xi’an, Shaanxi China; 5 Engineering Research Center of Personalized Anti-Aging Health Product Development and Transformation, Universities of Shaanxi Province, Xi’an, Shaanxi China; 6 Honghui Hospital, Xi’an Jiaotong University, Xi’an, Shaanxi China

**Keywords:** Gastric cancer, Biomarkers

## Abstract

Gastric cancer (GC) is the fifth most common cancer and the third leading cause of cancer death worldwide. Discovery of diagnostic biomarkers prompts the early detection of GC. In this study, we used limma method combined with joint mutual information (JMI), a machine learning algorithm, to identify a signature of 11 genes that performed well in distinguishing tumor and normal samples in a stomach adenocarcinoma cohort. Other two GC datasets were used to validate the classifying performances. Several of the candidate genes were correlated with GC tumor progression and survival. Overall, we highlight the application of feature selection approaches in the analysis of high-dimensional biological data, which will improve study accuracies and reduce workloads for the researchers when identifying potential tumor biomarkers.

## Introduction

Stomach cancer, or gastric cancer (GC), is the fifth most diagnosed cancer and the third leading cause of cancer death worldwide. The most common type of GC is stomach adenocarcinoma (STAD), which accounts for almost 90% of all GC cases^1^. Because the symptoms of GC can be mistaken for less serious problems like indigestion or heartburn, it is frequently misdiagnosed until the advanced stages. The outcome of GC treatment is generally poor, with a 5-year survival rate of nearly 30%^2^.

The non-invasive and low-cost advantages of diagnostic biomarkers prompt the researches of molecular biomarkers for the early detection of gastric cancer. However, the challenges in identifying promising biomarkers with exceptional discriminative performance are increasing^3^. As high-throughput technologies and machine learning methods advance, feature selection approaches for classification are being applied to cancer genomic studies^4^. The Cancer Genome Atlas (TCGA, https://www.cancer.gov/tcga) database, which includes 33 cancer types and over 20,000 primary cancer samples as well as massive gene expression data, has become one of the most widely used databases for cancer research^5–7^. In addition, the Genotype-Tissue Expression (GTEx, http://commonfund.nih.gov/GTEx) database establishes a reference resource of gene expression from ‘normal’ or disease-free tissues, which balances the sample size between tumor and normal groups and enlarges the samples when performing machine learning methods^8,9^. In general, there are tens of thousands of genes and far fewer samples than the number of genes in high-throughput sequencing data, forcing researchers to obtain appropriate biomarkers using machine learning heuristic algorithms. Feature selection has been used as a promising method to discover subsets of molecular markers that identify target classes of clinical cases^10^. The advantages of feature selection applied to high-throughput sequencing data are mainly manifested in two aspects: (1) the reduction of complexity through the elimination of relatively unimportant or redundant features, and (2) the improvement of classification accuracy and efficiency.

More efficient and robust feature selection methods are required to identify a small set of genes in order to improve classification performances. In this study, we combined two filter feature selection methods, limma and JMI algorithms, to identify key gene signatures for GC tumor and normal tissue discrimination. Specifically, we recruited the STAD cohort from the TCGA database as a test dataset, and two other GC datasets from NCBI-GEO DataSets were chosen as validation datasets. The expression alterations of the selected genes and clustering performance based on the selected genes were investigated. Besides, the biological application of the candidate genes were also analyzed.

## Materials and methods

### Data acquisition

As the testing dataset, STAD expression profiling was extracted from the total RSEM expected_count dataset (https://toil-xena-hub.s3.us-east-1.amazonaws.com/download/TCGA-GTEx-TARGET-gene-exp-counts.deseq2-normalized.log2.gz), which combines the cohort of TCGA, TARGET, and GTEx samples and can be downloaded from the UCSC xena website. The STAD dataset included 413 tumor samples (all from the TCGA database) and 210 normal samples (36 from the TCGA and 174 from the GTEx). For biological, the TCGA-STAD cohort’s survival dataset (https://gdc-hub.s3.us-east-1.amazonaws.com/download/TCGA-STAD.survival.tsv) and phenotype dataset (https://gdc-hub.s3.us-east-1.amazonaws.com/download/TCGA-STAD.GDC_phenotype.tsv.gz) were also acquired. Table 1 summarizes the clinicopathological characteristics of the 413 STAD tumor samples.

**Table 1:** Clinical characteristics of STAD cases.

	Group	Number
Age (year)	< 65 (year)	167
	≥ 65 (year)	214
	Not reported	3
	No information	29
Gender	Female	133
	Male	251
	No information	29
Race	Asian	84
	Black	12
	White	241
	Not reported	47
	No information	29
Tumor stage	Stage I	51
	Stage II	120
	Stage III	162
	Stage IV	37
	Not reported	14
	No information	29
OS (overall survival) status	Alive	225
	Dead	159
	No information	29
OS (overall survival) time (days)	Alive	724.42 ± 585.39
	Dead	439.06 ± 374.52
Total number		413

### Gene feature selection using the hybrid methods of limma and JMI

**Figure 1:**
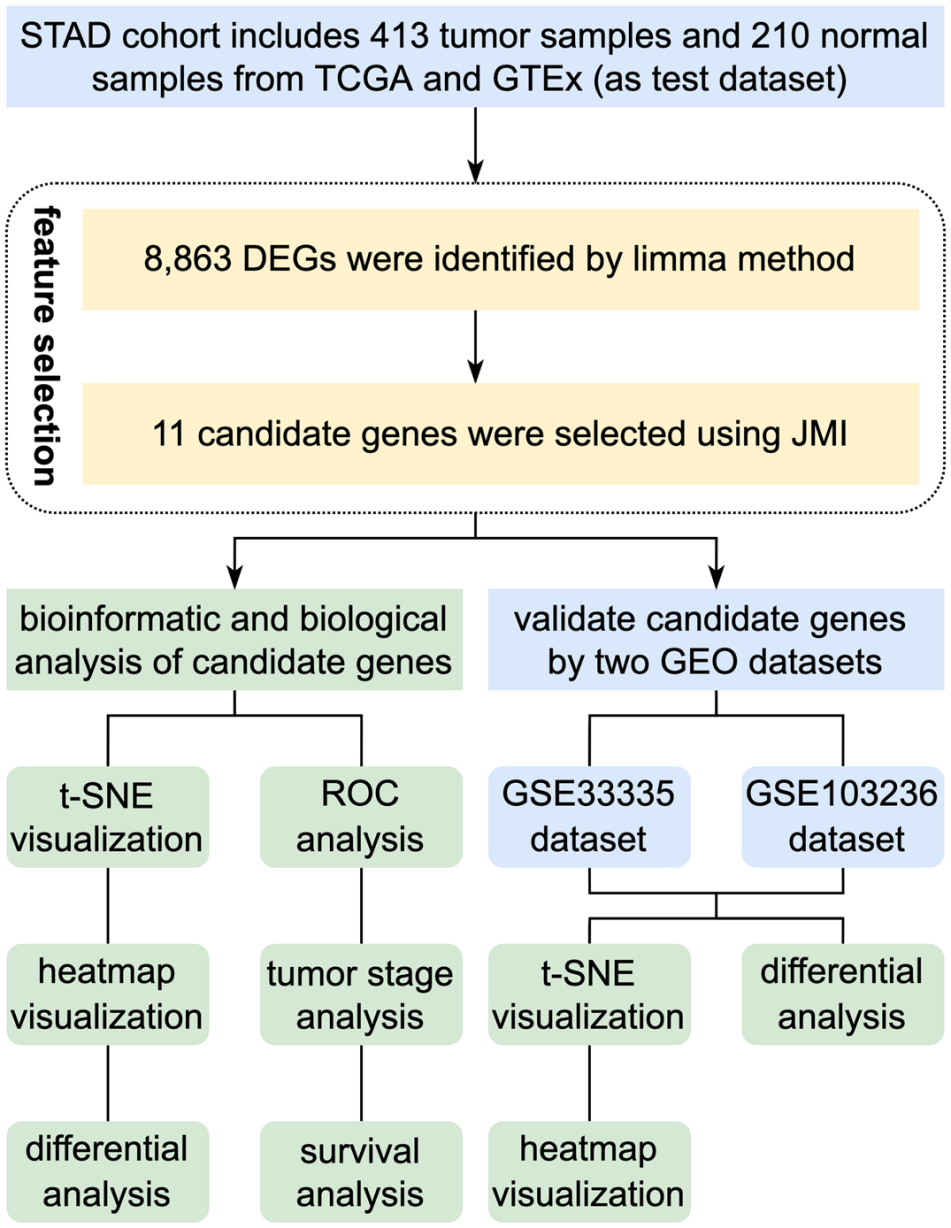
Design and workflow of the study. *STAD* stomach adenocarcinoma; *TCGA* The Cancer Genome Atlas, *GTEx* Genotype-Tissue Expression, *DEGs* differentially expressed genes, *JMI* joint mutual information, *GEO* Gene Expression Omnibus, *t-SNE* t-distributed stochastic neighbor embedding, *ROC* receiver operator characteristic.

Following the collection of gene expression profiles from a total of 623 samples (including 413 STAD tumors and 210 normal samples), we applied feature selection approaches to identify the gene biomarkers that are most relevant for classifying between tumor and normal groups. As shown in Fig. 1, the gene selection procedures mainly consist of two steps: gene filtering based on the limma package in R, and gene selection automatically using the joint mutual information (JMI) algorithm on condition of Python 3.8. We combined the filter and wrapper feature selection methods to obtain the gene subset with the highest classification efficacy and the least amount of redundancy.

Screening genes with the limma package: The limma package^11^ is based on R platform and aims to perform gene expression data analysis using linear models and differential expression functions, which can be used to perform comparisons between different groups. In the current research, we used the *lmFit* function in limma package to make model between tumor and normal groups, and the *makeContrasts* function was applied to build the contrast matrix. During the analysis, the value of logFC (fold change, FC) was required for gene filtering. The selection criterion for differentially expressed genes (DEGs) in this study was log|FC|> 1 and adj.*P* < 0.05 between tumor and normal groups.

JMI algorithm for removing redundancy and selecting the optimal gene subset: As the procedure for selecting DEGs based on the limma package did not take into account the interrelations among gene features, the obtained DEGs in the previous step may not optimal due to gene redundancy. As a result, removing redundancy genes from the selected total DEGs was necessary to improve not only the classification precision but also performance efficiency. In recent years, the feature selection method based on information theory, which aims to select the most relevant features from all features in order to reduce complexity of high-dimensional classification problems, has become the mainstream method^12^. The information gain (IG) method considers only the correlation between each sub-feature and the target classification separately, without taking into account the relationship between features. According to the principle of maximum dependency, maximum correlation and minimum redundancy based on mutual information, Peng et al. proposed the minimum redundancy Maximum relevance (mRMR) framework^13^, which has been applied for feature selection in many fields, including biological data. Equation 1 depicts the expression of the mRMR algorithm. Furthermore, the JMI algorithm^14,15^ provided a more comprehensive and widely used feature selection framework by taking mutual information between subset features and unselected features into account under IG-based classification conditions. The JMI algorithm was expressed as Eq. 2. Instead of focusing on the direct relationship in the mRMR framework, the JMI algorithm considers all mutual information between features, including the indirect correlation.In view of these, we used the JMI algorithm for feature selection based on the DEGs obtained from limma.

1 $$\alpha \left( {f_{i} } \right) = I\left( {f_{i} ;C} \right) - \frac{1}{\left| S \right|}\mathop \sum \limits_{{f_{s} \in S}} I\left( {f_{i} ;f_{s} } \right)$$

2 $$\alpha \left( {f_{i} } \right) = \mathop \sum \limits_{{f_{s} \in S}} I\left( {f_{i} ,f_{s} ;C} \right)$$

In the above two equations, *F*, *S* and *C* represent the total features, the selected features and the classification, respectively; while ${f}_{s}$ and ${f}_{i}$ mean the features belonging to *S* and *F\S*. $I\left({f}_{i},{f}_{s}\right)$ is the mutual information between ${f}_{s}$ and ${f}_{i}$, and $I\left({f}_{i},{f}_{s};C\right)$ is the mutual information between ${f}_{s}$,${f}_{i}$ and *C*. For the equation of $I\left({f}_{i},{f}_{s}\right)$, the larger the value of *I*, the stronger the correlation between ${f}_{s}$ and ${f}_{i}$.

In this study, we used both the two filter algorithms of limma and JMI as the hybrid method, to obtain key biomarkers for classifying tumor and normal tissues in gastric cancer. By this way, we could use as few gene features as possible to achieve better classification performances.

### Validation of the selected gene signatures with GEO datasets

After running the limma and JMI algorithms sequentially, we will obtain the candidate gene features for classifying tumor and normal samples in STAD cohort. We then validated the candidate genes in other two gastric cancer cohorts, including GSE33335 (https://www.ncbi.nlm.nih.gov/geo/query/acc.cgi?acc=GSE33335)^16,17^ and GSE103236 (https://www.ncbi.nlm.nih.gov/geo/query/acc.cgi?acc=GSE103236)^18,19^ from the NCBI-Gene Expression Omnibus (GEO) DataSets. The GSE33335 dataset contains gene expression profiling of 25 pairs of gastric tissues: gastric cancer tissues vs. matched adjacent noncancerous tissues. The GSE103236 dataset contains gene expression profiling of gastric adenocarcinoma (10 samples) and normal adjacent tissues (9 samples).

### Bioinformatic and biological analysis

T-SNE and heatmap analysis: The algorithms of t-distributed stochastic neighbor embedding (t-SNE)^20^ and bi-clustering analysis were performed in R using the "Rtsne" and “pheatmap” packages, respectively, to illustrate the distribution of GC tumor and normal samples based on the previously selected genes.

ROC analysis of candidate genes: To evaluate the diagnostic performance of candidate genes, we examined the specificity, sensitivity, and area under the curve (AUC) values obtained by using receiver operator characteristic (ROC) analysis in MedCalc software.

Identifying candidate genes associated with tumor stage and patient survival: The phenotype information including tumor stage and the survival information for STAD patients were derived from the TCGA database. Tumors are classified into four stages based on their stage status: I, II, III, and IV. We considered these genes to be stage-related biomarkers after discovering that changes in average gene expression were consistent with tumor stage progression. We used the R package "Survminer" to analyze and visualize the Kaplan–Meier curves of candidate genes based on their expression profiling for survival analysis.

## Results

### A set of 11 genes was identified as biomarkers to differentiate STAD tumor samples from normal samples

**Figure 2:**
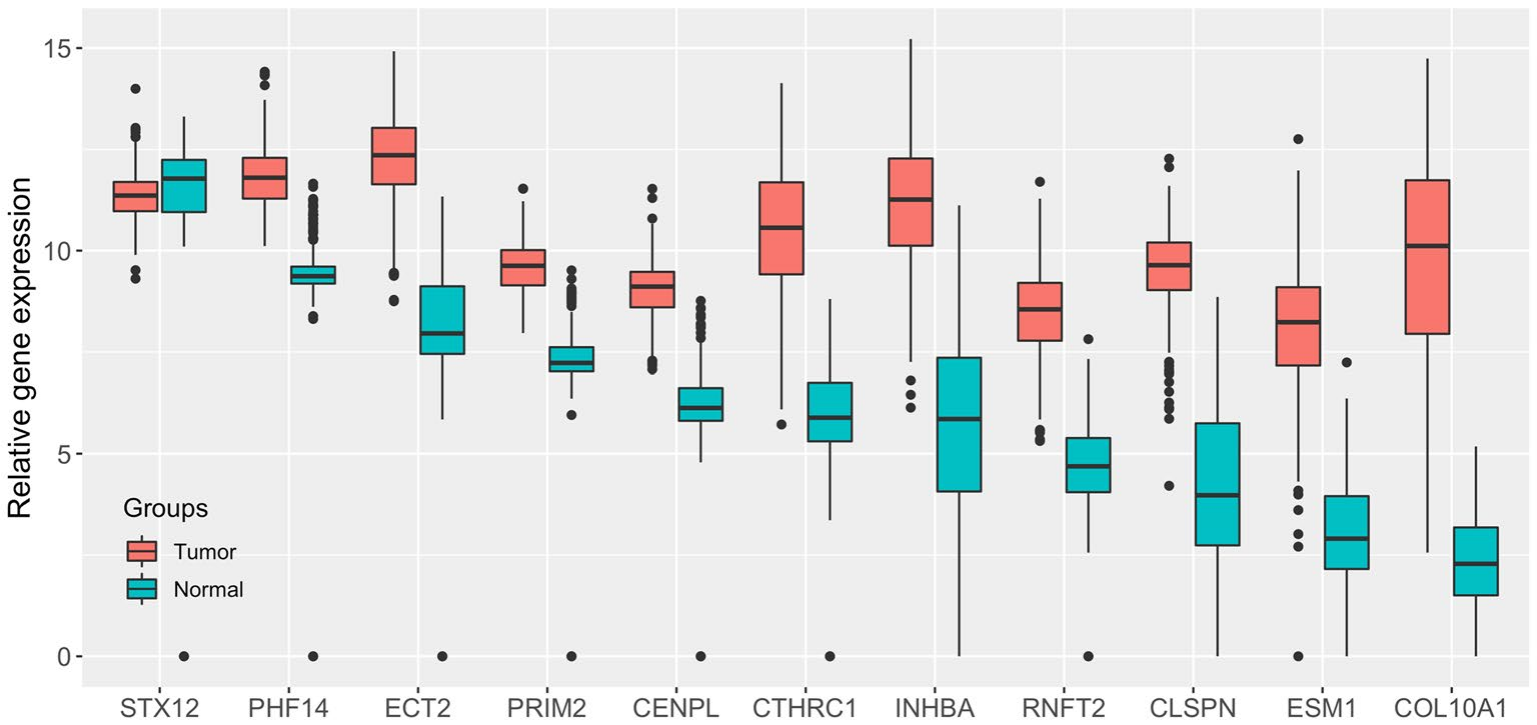
Relative expression levels of the 11 candidate genes in STAD tumor and normal samples. The data was obtained from the UCSC Xena website, and the boxplot displayed value ranges for each gene in two groups.

The original STAD dataset contains 60,499 gene identifiers profiles. After data preprocessing, we obtained expression profiles of 58,581 unique genes for each of the 623 samples. The “limma” algorithm was then used to initially selected the DEGs that differed between STAD tumor and normal samples. A total of 8,863 DEGs were screened using the criterion of log|FC|> 1 and adj.*P* < 0.05 between the two groups. The JMI algorithm was then used to obtain the optimal combination of gene features with maximum classification performance and minimum redundancy. Finally, a set of 11 genes were identified as candidate biomarkers for differentiating between tumor and normal groups among the 8863 DEGs, including STX12, PHF14, ECT2, PRIM2, CENPL, CTHRC1, INHBA, RNFT2, CLSPN, ESM1, and COL10A1. Only STX12 was down-regulated in tumors, while the other ten DEGs were all significantly up-regulated in tumors compared to the normal samples, as shown in Fig. 2.

### Performances of the 11 candidate genes in classifying tumor and normal groups

**Figure 3:**
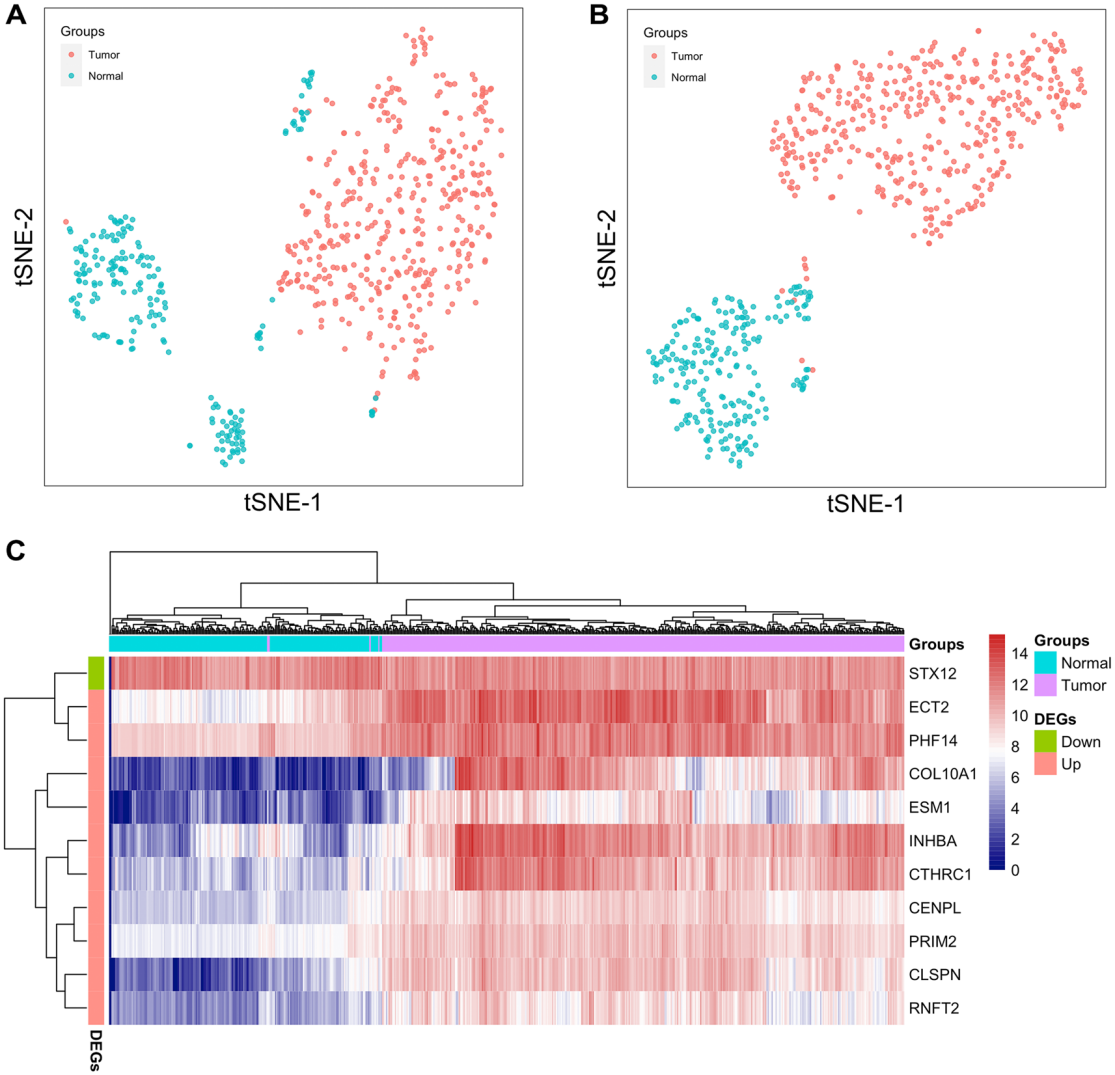
Classification of STAD tumor and normal groups. (**A, B**) T-SNE plots displayed the distribution of tumor and normal samples based on the 8,863 DEGs and the 11 candidate genes, respectively. (**C**) Bi-clustering heatmap of the 11 candidate genes and all 623 samples. DEGs: differentially expressed genes.

In this study, we performed t-SNE and bi-clustering heatmap to show the classification of STAD tumor and normal samples based on the DEGs profiling. Firstly, the t-SNE and heatmap visualization based on the 8,863 DEGs obtained from limma algorithm revealed a relatively distinct distribution between the two groups (Figs. 3A, S1). Further, after using the JMI method, we observed a more desirable discrimination model based on profiling of the 11 selected genes, with only four (4/413) tumor samples distributed concordantly into the normal group in both t-SNE and heatmap analysis (Fig. 3B,C). The results indicated that the combination of limma approach and JMI algorithm improved the accuracy and efficiency in classifying different groups. Finally, the ROC analysis revealed generally excellent results when the selected genes were used as diagnostic biomarkers. Specifically, the AUCs of the ten up-regulated genes in tumors ranged from 0.983 to 0.990, whereas the diagnostic performance of the down-regulated gene STX12 in tumors was less than optimal, with AUC = 0.615, as shown in Fig. S2A.

### Validation of the 11 selected gene signatures in two GEO datasets

**Figure 4:**
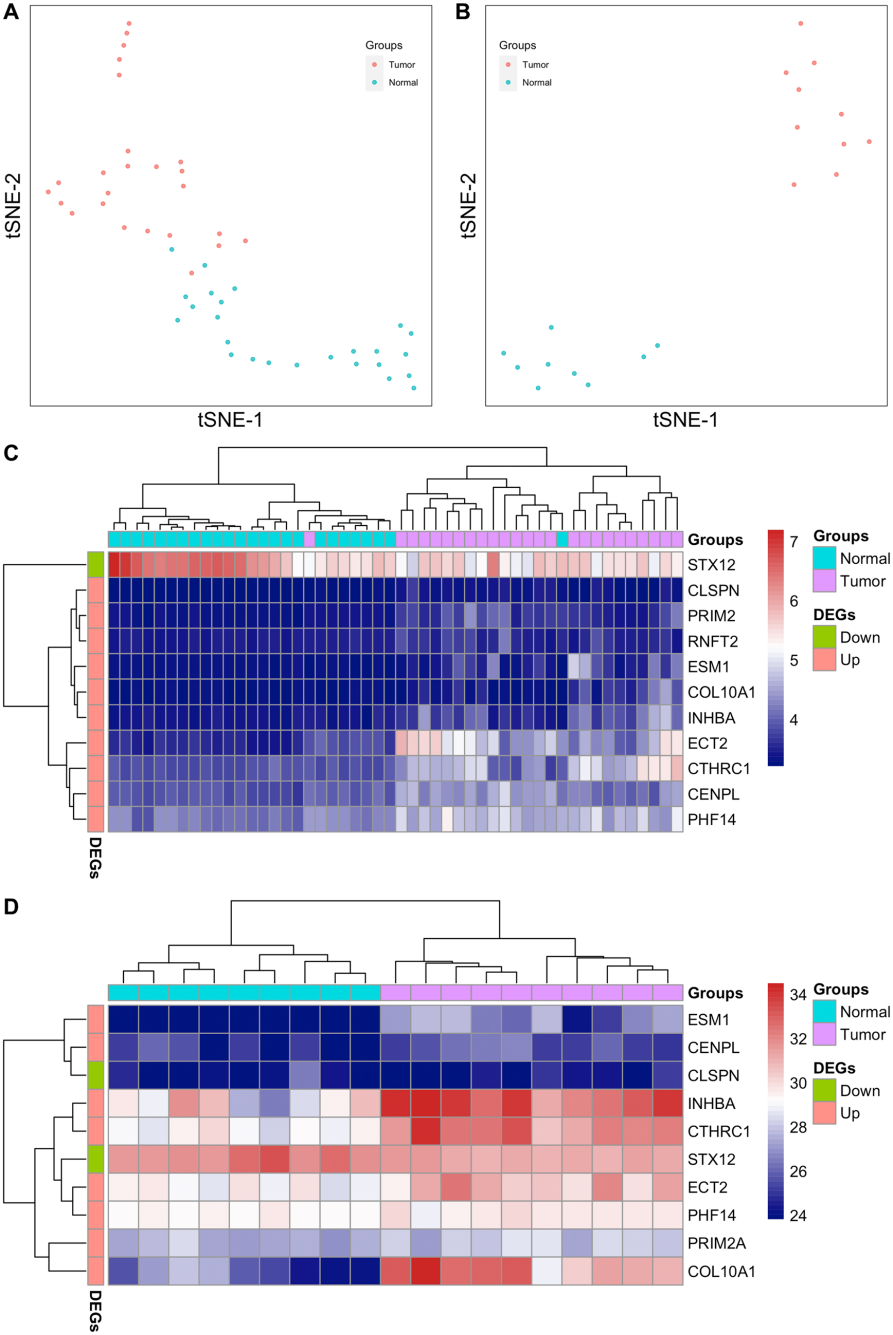
Classification between gastric tumor and normal tissues. (**A**) T-SNE plots displayed the distribution of tumor and normal samples based on the 11 candidate genes in the GSE33335 dataset. (**B**) T-SNE plots displayed the distribution of tumor and normal samples based on the ten candidate genes in the GSE103236 dataset. (**C**) Bi-clustering heatmap of the 11 candidate genes and 50 samples in the GSE33335 dataset. (**D**) Bi-clustering heatmap of the ten candidate genes and 19 samples in the GSE103236 dataset.

As described in the method, we chose two datasets of gastric cancer from the GEO databases to confirm the expression pattern and classifying performance of the previously selected 11 genes. In the GSE33335 dataset, we found a consistent expression pattern, in which the expression of ten of the 11 genes increased while the expression of STX12 decreased in tumor compared to normal tissues (*P* < 0.001 in two-tailed paired-sample t test, Fig. S3A). In the GSE103236 dataset, the expression profiling contains about 45,000 gene IDs, while the expression information of gene RNFT2 was not included in the total gene expression profiling. Therefore, we analyzed the remaining ten candidate genes in this dataset. In general, the expression patterns of these ten genes were almost identical to the previous results, with the exception that CLSPN expression was slightly lower in tumor tissues than in normal tissues, but this difference was not statistically significant (*P* = 0.81). Similarly, STX12 expression was significantly reduced while the expression of the other eight genes increased in tumor tissues (*P* < 0.05 in two-tailed t test, Fig. S3B). We then investigated the classifying abilities of the selected gene signatures in the two datasets. As shown in Fig. 4, t-SNE visualizations and heatmaps based on the gene signatures produced satisfactory discrimination clusters between tumor and normal groups. The AUCs of these 11 genes ranged from 0.806 to 0.979 in the GSE33335 dataset. The performance of gene CLSPN in the GSE103236 dataset was poor, with AUC = 0.528; and AUCs of the other nine genes ranged from 0.789 to 1.000. Taken together, these findings support the promising application of feature selection approaches in the processing of high-throughput biological data.

### Identifying gene biomarkers associated with stage and survival in STAD tumors

**Figure 5:**
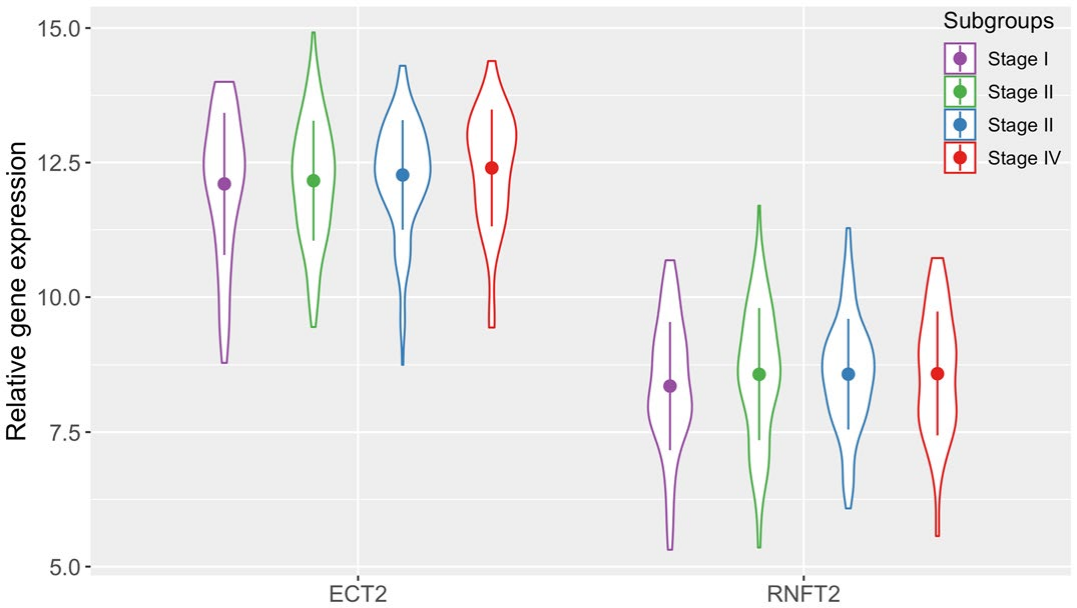
Relative expression levels of ECT2 and RNFT2 in tumor subgroups at virous stages. The violin boxplots depicted value ranges of the two genes, with the dot in each plot representing the average value.

Next, we investigated whether the expression profiles of the candidate genes could reflect tumor progression or predict the prognosis. Table S1 summarized the relative gene expression of the 11 candidate genes in four subgroups according to tumor stage. Specifically, in the ten up-regulated genes, we discovered that the relative expression levels of two genes, ECT2 and RNFT2, gradually increased with the progression of stage status, as shown in Fig. 5. The average values of ECT2 in four subgroups were 12.10, 12.16, 12.27, 12.40, respectively; and the average values of RNFT2 in four subgroups were 8.36, 8.57, 8.58. 8.59, respectively. Despite the fact that gene expression levels were only moderately or slightly different among the four subgroups, the results demonstrated that these two genes may have the potential to reflect tumor progression to some extent.

**Figure 6:**
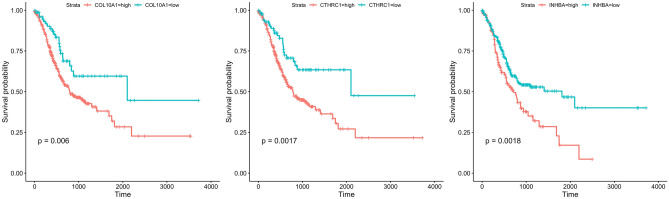
Kaplan–Meier survival curves based on COL10A1, CTHRC1, and INHBA expression levels. The cut-off values classified gene expression as high (high) or low (low). The horizontal axis represents survival time (days), and the vertical axis represents overall survival rate.

The overall survival (OS) Kaplan–Meier plot also revealed significantly different prognostic outcomes based on gene expression level. Higher levels of genes including COL10A1, CTHRC1, and INHBA were associated with poor survival probabilities in STAD patients (*P* < 0.01, Fig. 6).

## Discussion

While the development and generation of high-throughput technologies and omics data have improved our understanding complex biological characteristics such as tumors, brains, and developmental systems, they have also created significant technical challenges during the analysis. Typically, researchers identify differentially expressed/abundant molecules (genes, proteins, or metabolites) between tumor and control groups as preliminary results for biomarker analysis. We must accept the fact that hundreds or even thousands of DEGs between two groups were always detected in the high-dimensional gene expression datasets. As a result, in addition to traditional methods, more approaches are required to efficiently and accurately select the key gene signatures that could classify the disease between normal samples^21,22^. In this study, we combined two approaches, limma and JMI algorithms, and finally identified a set of 11 genes with powerful discrimination effects between STAD tumor and normal tissues. Remarkably, additional independent validation with two GC datasets confirmed the expressed alterations as well as the classifying performance of the chosen gene sets.

Some candidate genes have been linked to gastric cancer. COL10A1, a member of the collagen family and the main matrix component, is high expressed in gastric cancer compared to the normal tissues and an independent predictor of poor overall survival^23^. On the mechanism, COL10A1 was confirmed to be a potential inducer of epithelial-to-mesenchymal transition (EMT) and could regulate the migration and invasion of GC cells^24^. CTHRC1 is known to be involved in tissue remodeling processes and closely associated with carcinogenesis and metastasis in solid tumors^25^. CTHRC1 has been shown to prompt gastric cancer metastasis via HIF-1α/CXCR4 signaling pathway^25^, and to be a common diagnostic and prognostic biomarker in six different human cancer subtypes, including STAD^26^. Highly expression of INHBA and activation of TGF-β signaling pathways were observed in GC tissues, and INHBA gene silencing inhibited the GC progression by inactivating the TGF-β signaling pathway^27^. INHBA may also be an optimally reliable biomarker for diagnosing GC and lymph-node (LN) metastasis^28^. Consistent with the above reports, we discovered that the expressions of COL1A1, CTHRC1, and INHBA were significantly up-regulated in tumor tissues in the TCGA-STAD cohort and two GEO gastric cancer datasets. Furthermore, high expression levels of the three DEGs were associated with significantly poor survival probabilities in tumor patients (*P* < 0.01). Besides, we also discovered that the average expressions of two genes, ECT2 and RNFT2, increased concordantly with the tumor stage progression. Recent studies reported that upregulation of ECT2 predicted adverse clinical outcomes and increased 5-fluorouracil (5-Fu) resistance in GC patients^29^, and was associated with transcriptional program of cancer stem cells (CSCs)^30^. So far, studies on the roles of RNFT2 in cancer research is relatively rare. A recent study demonstrated that tissue RNFT2 expression levels are associated with peritoneal recurrence and poor prognosis in GC^31^. Taken together, our results and other related findings suggest that the five upregulated genes mentioned above could be potential targets for GC research.

In contrast to the upregulated DEGs, we discovered that STX12 expression was significantly and consistently lower in tumor compared to normal tissues in all three GC cohorts. STX12 is a SNARE protein that mediates vesicle fusion at endosomes and functions as a new component of the α-granule biogenesis machinery^32^. STX12 was upregulated through the ROS/STAT3/NFE2L1 axis in hepatoma cells, and it was a key downstream effector of NFE2L1 in modulating hepatoma cell invasiveness^33^. So far, there has been no report on the roles of STX12 in gastric cancer, which may necessitate further studies.

## Conclusion

In conclusion, our study identified a small set of genes (11 candidate genes including STX12, PHF14, ECT2, PRIM2, CENPL, CTHRC1, INHBA, RNFT2, CLSPN, ESM1, and COL10A1) that could be used to distinguish gastric cancer from normal tissues in TCGA cohort by using the combination feature selection methods of limma and JMI. Meanwhile, the classification performance of the candidate genes was further validated in other two gastric cancer cohorts in GEO datasets. Moreover, we explored that several candidate genes involved in gastric cancer progression and prognosis. We highlighted the application of machine learning, particularly feature selection approaches, in the analysis of high-dimensional biological data for discovering valuable biomarkers, which will improve accuracies and reduce workloads for the researchers when identifying potential biomarkers of tumors.

## Supplementary Information

Supplementary Information 1.

Supplementary Information 2.

Supplementary Information 3.

Supplementary Information 4.

Supplementary Information 5.

## Data Availability

The datasets analysed during the current study are available in the FigShare repository, https://figshare.com/articles/dataset/Gene_expression_data_in_gastric_cancer/19733347.
