# Supplementary material for: Identification of key biomarkers for STAD using filter feature selection approaches
Source: Sci Rep. 2022 Nov 18;12:19854. doi: 10.1038/s41598-022-21760-w (PMC9674689; doi:10.1038/s41598-022-21760-w)
Supplement: Supplementary file 1 — Supplementary Information 1. [file 41598_2022_21760_MOESM1_ESM.docx]

**Fig. S1** Bi-clustering heatmap of the 8,863 DEGs in 623 samples.

**Fig. S2** ROC curves of 11 candidate genes in discriminating between tumor and normal samples. **A** In TCGA cohort, AUC of gene STX12 was 0.615, and AUCs of the other ten genes ranged from 0.983 to 0.990. **B** In GSE33335 dataset, AUCs of these 11 genes ranged from 0.806 to 0.979. **C** In GSE103236 dataset, AUC of gene CLSPN was 0.528, AUCs of the other nine genes ranged from 0.789 to 1.000.

**Fig. S3** Relative expression levels of 11 candidate genes in two GEO datasets. **A** GSE33335 dataset. **B** GSE103236 dataset. In this dataset, expression of RNFT2 was not annotated in the total gene expression profiling, therefore, the boxplot displayed ten genes here.
