# Supplementary figures and images for: Identification of key biomarkers for STAD using filter feature selection approaches
Source: Sci Rep. 2022 Nov 18;12:19854. doi: 10.1038/s41598-022-21760-w (PMC9674689; doi:10.1038/s41598-022-21760-w)

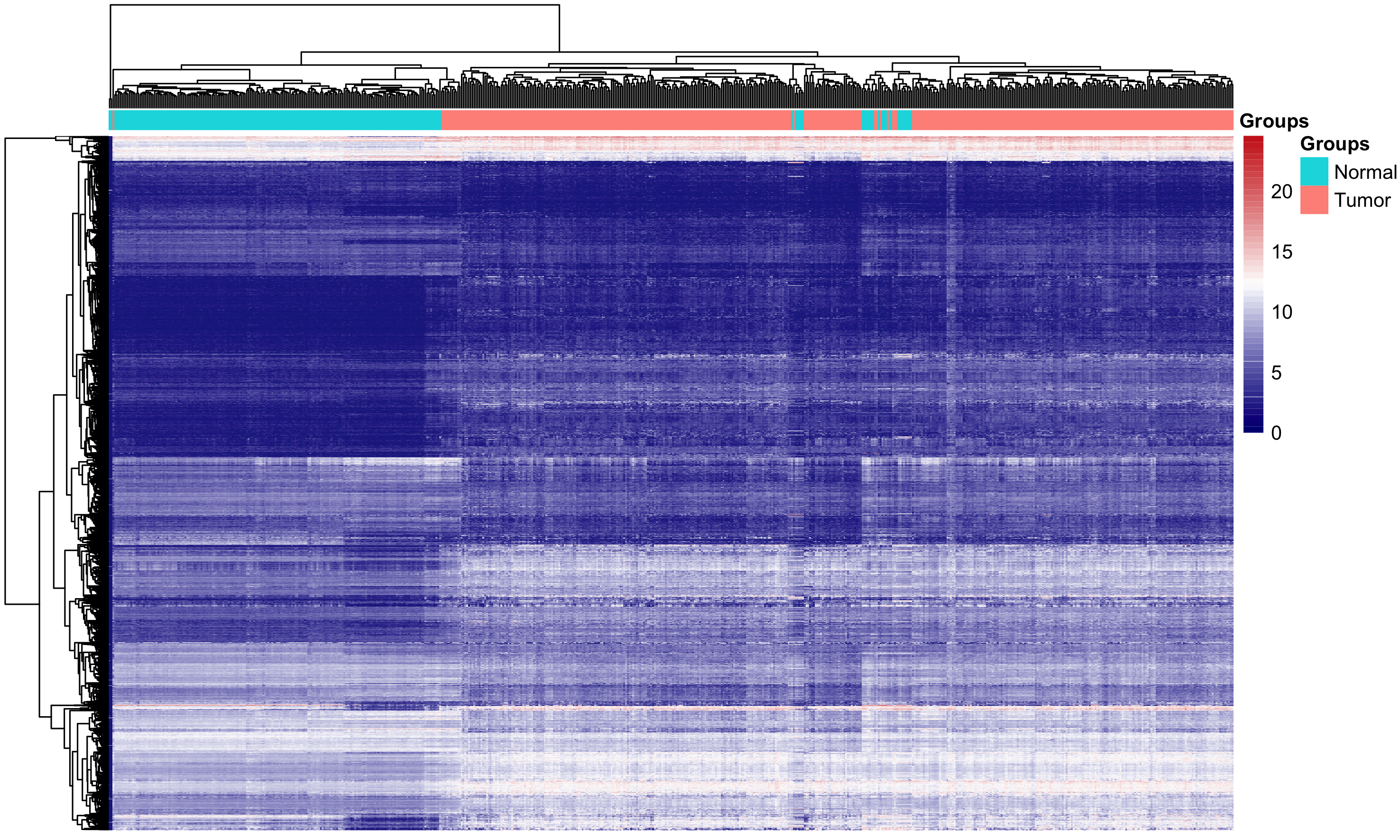

Supplement: Supplementary file 2 — Supplementary Information 2. [file 41598_2022_21760_MOESM2_ESM.tif]

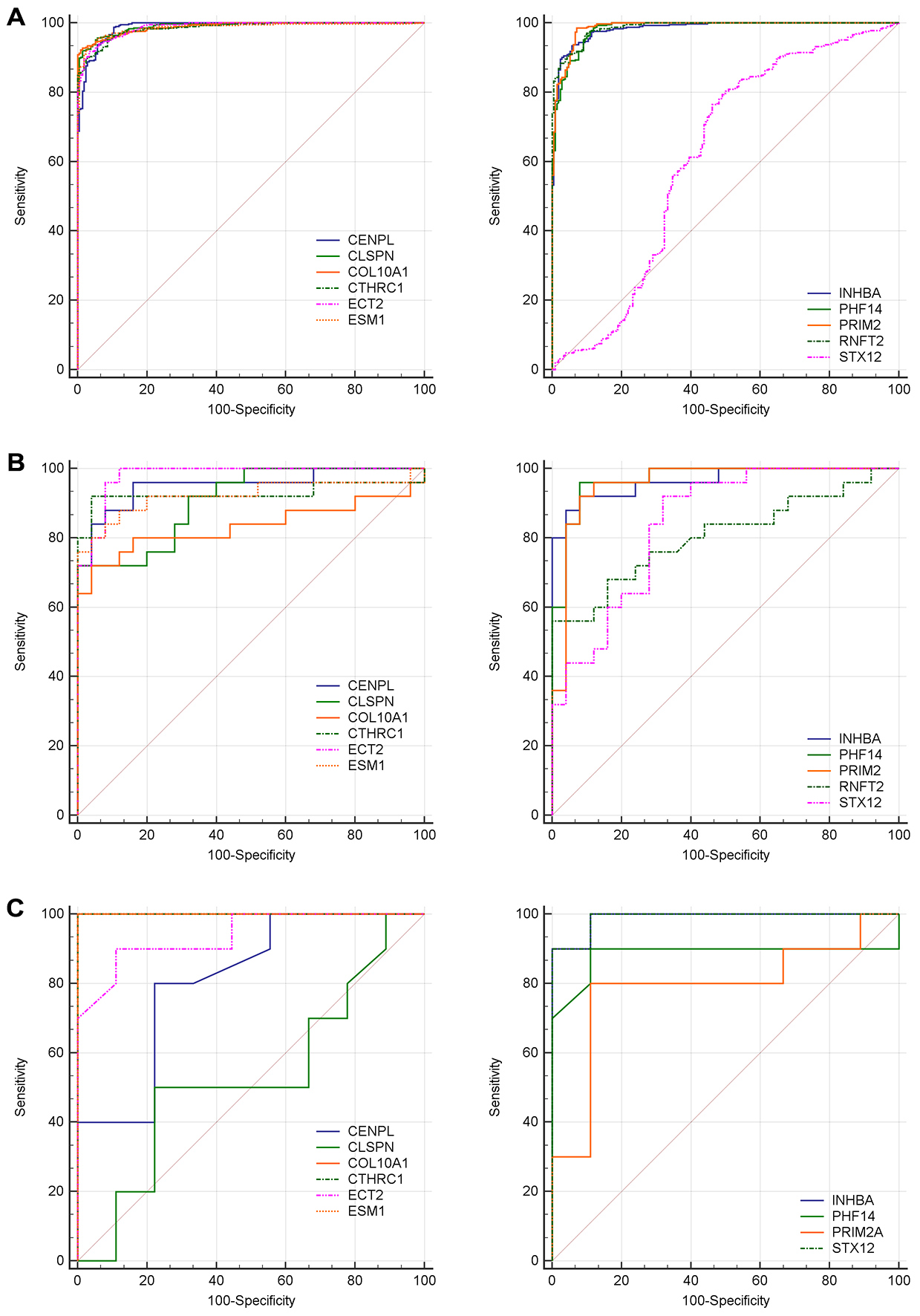

Supplement: Supplementary file 3 — Supplementary Information 3. [file 41598_2022_21760_MOESM3_ESM.tif]

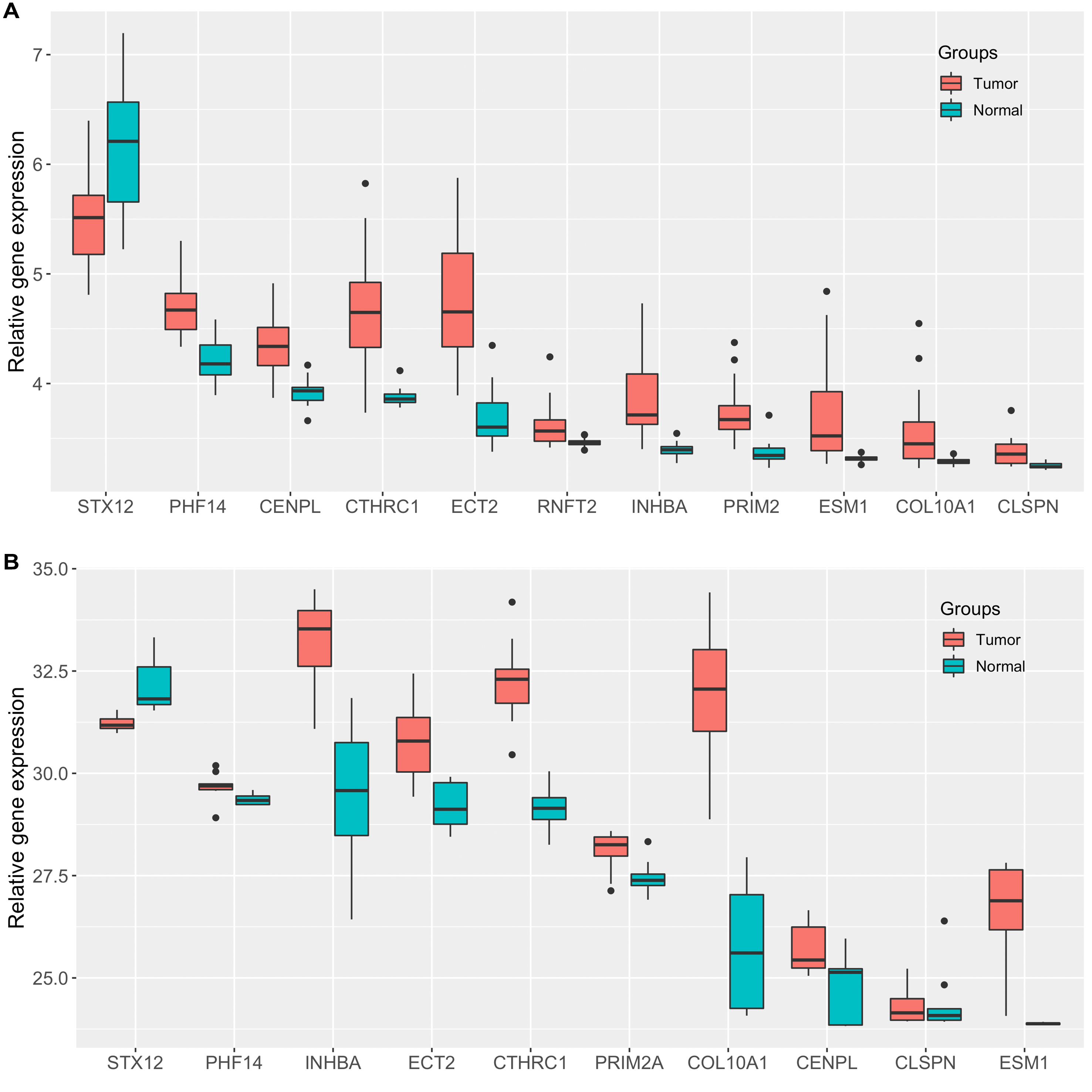

Supplement: Supplementary file 4 — Supplementary Information 4. [file 41598_2022_21760_MOESM4_ESM.tif]
