# Supplementary material for: Identification of key biomarkers for STAD using filter feature selection approaches
Source: Sci Rep. 2022 Nov 18;12:19854. doi: 10.1038/s41598-022-21760-w (PMC9674689; doi:10.1038/s41598-022-21760-w)
Supplement: Supplementary file 5 — Supplementary Information 5. [file 41598_2022_21760_MOESM5_ESM.docx]

**Table S1. Relative gene expression of the 11 candidate genes in four subgroups according to tumor stage**

|  | Stage I | Stage II | Stage III | Stage IV |
| --- | --- | --- | --- | --- |
| STX12 | 11.25 | 11.35 | 11.31 | 11.33 |
| PHF14 | 11.57 | 11.85 | 12.01 | 11.58 |
| ECT2 | 12.10 | 12.16 | 12.27 | 12.40 |
| PRIM2 | 9.53 | 9.65 | 9.59 | 9.55 |
| CENPL | 9.03 | 9.00 | 9.01 | 9.04 |
| CTHRC1 | 9.76 | 10.76 | 10.43 | 10.52 |
| INHBA | 10.46 | 11.40 | 11.14 | 11.20 |
| RNFT2 | 8.36 | 8.57 | 8.58 | 8.59 |
| CLSPN | 9.37 | 9.58 | 9.63 | 9.51 |
| ESM1 | 7.97 | 8.18 | 8.05 | 8.34 |
| COL10A1 | 8.10 | 10.25 | 9.53 | 9.87 |
